# Dynamics of regional knowledge creation and inter-city R&D collaboration in China: evidence from an empirical agent-based simulation model

**DOI:** 10.1007/s00168-025-01402-5

**Published:** 2025-07-12

**Authors:** Martina Neuländtner, Thomas Scherngell, Xielin Liu, Xiaohui Ji, Xuechen Ding, Manfred Paier

**Affiliations:** 1 AIT Austrian Institute of Technology GmbH, Giefinggasse 4, 1210 Vienna, Austria; 2 School of Economics and Management, University of Chinese Academy of Sciences, 80 Zhongguancun East Rd., Beijing, 100190 China; 3 College of Business Administration, Capital University of Economics and Business, 121 Shoujingmao S Rd, 100071 Beijing, China; 4 Business School, Beijing Technology and Business University, 33 Fucheng Road, 100048 Beijing, China

**Keywords:** O31, R11, C63, R58

## Abstract

The objective of this study is to simulate the dynamic relationships between regional knowledge creation and inter-city R&D collaboration in China. We contribute to the scarce literature using empirically driven agent-based simulation for modelling regional knowledge creation. We provide original evidence for China creating a better understanding of the underlying micro-dynamics that shape regional knowledge creation rather than on aggregate levels (countries or larger regions). We demonstrate that collaboration produces higher knowledge gains at the system level, but benefits differ across Chinese cities. Moreover, technology-specific knowledge gains from collaborations depend on place-based factors locally moderating collaboration benefits in specific technologies.

## Introduction

Over the last decades, China has followed a unique path of growing economic and technological maturity driven by an unprecedented transformation from a low-cost to an innovation-driven economy. This shift was accompanied by a set of new policy measures paving the way for a systemic change in the Chinese science and innovation system (Frietsch 2020). The rapid development and growing maturity of the regional innovation systems resulted in the comparably strong spatial concentration of innovative activities, fundamentally driven by agglomeration forces, linked to population, industrial specialisation, and infrastructure endowment (Crescenzi et al. 2017). While, on the one hand, these agglomeration factors highlight the importance of spatial proximity for knowledge creation, on the other hand, strengthening of research and development (R&D) collaborations between regions is increasingly stressed to facilitate access to new specific knowledge or capabilities that are located further away in geographical space (Scherngell 2013); and potentially, by this, decrease regional socio-economic disparities. This emphasises the increasingly networked character of knowledge creation processes, characterised by a dense web of inter-organisational knowledge interactions in the innovation process.

In Western countries, particularly countries of the European Union (EU), inter-regional R&D collaborations are well-established vehicles of spatial knowledge diffusion and are an integral part of the national and regional innovation systems. Recently, the Chinese government has announced a set of policy measures that point to the crucial importance of inter-regional R&D collaborations between cities to contribute to integrating regional innovation systems (Jiang et al. 2017). For instance, lately, the ‘Made in China 2025’ (MIC2025) initiative stresses the role of universities and research organisations, innovation alliances and collaboration with industry. With China’s recent shift to an innovation-driven economy and with its enormous economic growth, accompanied by various policies and strategies aiming for China to become an international innovation leader, China’s role in global innovation processes has attracted considerable attention. This spurred the research interest in understanding the driving forces behind this exceptional economic and technological catching-up process. In this respect, studies emphasising the role of regions (as subnational spatial entities) as loci of knowledge creation and technological progress focus on the emergence of innovation systems and the effect of the openness of such local systems to cross-regional interactions (see, e.g. Asheim et al. 2016).

Up to now, there is only scarce research investigating drivers of regional knowledge creation in China, in particular for geographical breakdowns below the province level, but also in terms of relating regional knowledge creation to inter-city R&D collaboration networks. Moreover, most evidence provided so far—also for other territories than China (e.g. the EU or the US)—is done at an aggregate level of regions, neglecting the underlying micro-dynamics that play a role in shaping regional knowledge creation capabilities. In this study, we contribute to closing these research gaps by the mobilisation of novel data on research and innovation activities at a very detailed geographical level while accounting for micro-dynamics at the regional level following a simulation approach, namely by means of Agent-Based Modelling (ABM). The objective is to develop and apply an empirical ABM for regional knowledge creation in China, specifically shifting attention to simulate the dynamic relationships between regional knowledge creation and inter-city R&D collaboration. Importantly, we initialise, calibrate, and validate the empirical simulation model employing large-scale empirical data. The model features over 42 thousand agents active in 37 industry sectors—being representative for 289 Chinese regions (285 cities and four municipalities) in their characteristics.

So far, empirical studies following this path are often conducted at a fairly aggregate level (e.g. provinces) based on exploratory spatial data analysis and spatial econometric models or focus on single regions or sectors by means of detailed case studies (see, e.g. Scherngell and Hu 2011). In contrast, we propose a unique and widely unexplored approach in regional science, namely ABM, to explore the complex interplay between inter-regional R&D collaboration and regional knowledge creation (with the exception of some recent works, e.g. Wang 2018; Sebestyén and Varga 2019; Neuländtner 2020). By this, we depart from previous research by explicitly accounting for the heterogeneous nature of regions with respect to their diverse research actors, institutional characteristics, sectoral distributions, and policy targets. ABM is a computer simulation technique where entities are modelled and implemented in terms of agents and their individual behaviours (e.g. Conte et al. 1998). The core idea of the ABM approach is that macro phenomena emerge from the individual agents’ behaviour (Schillo et al. 2000). Together, individual agents and the simulation environment constitute a ‘bottom-up’ complex system (Gu et al. 2011). This allows us to conduct simulation experiments in a controlled environment by inducing specific alterations to individual-level behaviours of actors and analyse the resulting output changes at an aggregate, system level. In our focus on inter-city R&D collaboration networks, we specifically shift attention to simulation scenarios comparing knowledge creation mechanisms under an increased intensity of R&D collaboration to the current state of knowledge creation (baseline scenario). In a policy context, the study highlights important pointers to potential intervention mechanisms to be considered for the Chinese innovation policy.

The remainder of this study is organised as follows. Section 2 shifts attention to the theoretical background, reviewing the main considerations of the literature on R&D collaboration networks and regional knowledge creation and introducing the Chinese innovation and regional policy background. Section 3 follows with a more general introduction of the ABM approach, before Sect. 4 emphasises the theoretical model conception of the ABM for regional knowledge creation. Then, Sect. 5 outlines the specific empirical model specification, starting with the empirical initialisation and calibration before coming to a baseline scenario re-creating the current status of knowledge creation across Chinese cities. Section 6 discusses the simulation results, comparing a scenario of increased collaboration to the baseline scenario along specific critical analytical dimensions (total knowledge gains, spatial distribution, technological dynamics). Section 7 closes with a summary of the main results, some conclusions and policy implications and some ideas for future research.

## Theoretical background

There is a wide literature debating the complex relationships between regional knowledge creation and R&D collaboration networks (see Scherngell 2013). In what follows, we initially discuss the theoretical considerations on the relationship between R&D collaboration and knowledge creation. Afterwards, we elaborate on this debate in the Chinese context, pointing to the increasing importance of Chinese innovation policy in our focus on R&D collaboration.

### Regional knowledge creation and R&D collaborations

The increasingly complex nature of innovation processes demands access to diverse pieces of knowledge that are often located outside the regional knowledge base. In modern R&D activities, collaborations between researchers affiliated with organisations of different types have become the norm rather than the exception (Powell and Grodal 2005, Malerba and Vonortas 2009, Cantner and Graf 2011, Scherngell 2021, among others). They are considered a determining factor for the success and efficiency of knowledge production and innovation. In particular, in times of increasing globalisation and rapidly changing demand conditions, knowledge-producing organisations are confronted with higher uncertainty and risks in their R&D activities. Collaborative arrangements are considered promising instruments in such highly dynamic and complex knowledge-based economies, being confronted with rising costs and uncertainty in knowledge creation processes.

This spurs the interest in such R&D collaboration networks, not only from a scientific but also from a policy perspective, since they can serve as channels for transmitting new knowledge—also over larger geographical distances (e.g. Breschni and Cusmano 2006, Autant‐Bernard et al. 2007, Scherngell and Barber 2009; Scherngell and Lata 2013; Wanzenböck and Piribauer 2018; Neuländtner and Scherngell 2022). They are assumed to be important for overcoming geographical barriers to accessing external knowledge. In the long-term they support the reduction of regional inequalities in the distribution of knowledge and avoidance of regional lock-ins to an increasingly obsolete technological trajectory (Cantwell and Iammarino 2005). It is assumed that R&D collaborations are able to moderate and partly reduce the strong geographical localisation of knowledge flows that is driven by the sticky nature of knowledge (Asheim and Isaksen 2002), in particular of tacit knowledge that is specifically relevant for creating new knowledge and, accordingly, innovation (Maggioni and Uberti 2011; Scherngell 2013).

Against this background, cross-region R&D collaboration networks have attracted increasing interest, in particular from an empirical perspective in the geography of innovation literature (Feldman and Kogler 2010). New large-scale datasets, but also methodologies combining traditional spatial data analytic methods with approaches from network science, have enabled several more comprehensive works investigating the structure and dynamics of such R&D collaboration networks (see Scherngell 2021 for an overview). In the latter context, it is evidenced that specific forms of separation between regions shape the intensity of R&D collaboration between them, most intuitively geographical distance, but also technological, cultural and institutional separation effects, among others. While these drivers for R&D collaboration are relatively well researched in the recent past, there is much scarcer robust empirical evidence on the various effects of the embedding in such collaboration networks on regional knowledge creation. Whereas, for the European case, some studies have employed a spatial econometric perspective to identify the influences of collaboration networks on regional knowledge creation capabilities (see, e.g. Wanzenböck and Piribauer 2018; Neuländtner and Scherngell 2022), studies taking a simulation perspective are scarce (Neuländtner 2020). We develop and employ an empirical ABM to explore relationships between R&D collaborations and regional knowledge creation, applied to China as an extremely interesting case given its economic rise over the past four decades. In what follows, we discuss in some more detail the Chinese context—from a perspective of regional innovation and innovation policy—to span the investigated landscape for the empirical model derived in Sect. 5.

### Regional innovation in China and policy background

Based on the theoretical and empirical insights discussed in the previous subsection, the promotion and support of regional innovation capability have become core policy issues in current STI policies, not only in the US and Europe (e.g. the EU Framework Programme) but also in China. In the Chinese context, one has to consider the significant decentralisation processes that started in the 1980s to understand the context, making regions much more autonomous. For a long time, regional decentralisation seemed the key strategy for regional development, as this promoted regional competition and provided incentives for regional officials to create and allocate resources for economic development. Since then, regions have begun to compete with each other, which also gave them the motivation for a more entrepreneurial state. However, in the last decade, it is believed that this decentralisation may harm the development as it may cause over-competition and market disintegration. As regions have different resource endowments and development strategies, specific regions lead ahead after some years, while others lag behind, however some show potential to catch-up.

In order to narrow the development gap, both, developed and emerging economies have tried to establish dedicated policy strategies, for example, in the EU, smart specialisation. In China, more and more similar policy initiatives have targeted this issue over the last decade, including efforts to foster cross-regional R&D collaboration. Specifically, during the period of the 13th Five-Year Plan (2016–2020), one of the main foci has been to strengthen the cooperation between regions in China. In this respect, major regional development strategies can be summarised into three initiatives^1^: Firstly, fostering the so-called strategic city clusters based on geographic proximity of important innovative cities, including the Beijing-Tianjin-Hebei cluster and the Guangdong-Hongkong-Macao greater bay area, aiming to build these agglomerations into world-class innovation and technology hubs. *Secondly*, strengthening cooperation among cities along the upstream and downstream coasts of important river basins, such as Yangtze River Economic Belt, Pearl River-West River Economic Belt, Huaihe River Economic Belt and Han River Ecological Economic Belt. This strategy aims to use important river basins as links to connect coastal and inland cities, thereby improving the level of openness of underdeveloped inland cities and enhancing the convergence and coordination among regions. *Thirdly*, boosting the collaboration among inter-provincial boundary regions, such as the Chengdu-Chongqing Economic Circle, the golden triangle area of the Yellow River in Shanxi, Shaanxi and Henan Provinces, Guangdong-Guangxi Interprovincial Pilot Cooperation, and Hunan-Jiangxi Interprovincial Pilot Cooperation. The blueprint has provided important strategic opportunities for the development of cities far from the economic growth poles in the provinces and is also an active attempt to improve policy coordination between administrative regions.

In this light, Chinese scholars have devoted increasing attention to inter-regional collaboration in China as a key driver for regional innovation and a vehicle for regional diversification. For instance, Tang and Guan (2021) found in the Chinese context that non-local connections enable actors to gain access to complementary and diversified external sources of knowledge, thus enhancing interaction with local knowledge assets and improving innovation output. Wang and Zhang (2019) proposed that knowledge pieces received from outside regions can be used to expand the local knowledge base and provide new momentum for development, which is often treated as a possible escape from spatial lock-in for backward regions. However, some scholars have found that the effect of such inter-regional cooperation is not always positive. Ye and Xu (2021) argue that external knowledge sourcing is risky and costly, which requires actors to identify and absorb relevant knowledge. In this case, less developed areas have to bear the negative effect of knowledge redundancy on innovation because they cannot fully absorb external knowledge. Moreover, Zhang et al. (2016) found that most technologies are transferred to developed provinces. Liu et al. (2022) confirmed the existence of the siphon effect in China's regional development and found that developed regions can gather more high-quality human and capital elements, thereby accelerating the loss of innovation elements in less developed areas. That is to say, inter-regional cooperation can accelerate the flow of knowledge elements and high-quality talents from underdeveloped areas to more developed regions, widening the innovation gap between them (Tang and Guan 2021). Hence, especially for lagging regions, it is necessary to implement effective policies to reduce the draining impact of inter-regional cooperation, given China’s current development. Nevertheless, all in all, the positive effect of inter-regional cooperation on innovation output cannot be ignored.

However, up to now, there is no systematic evidence of the different effects of cooperation on regions and the underlying mechanisms of action. This study contributes to this debate from a comprehensive, empirically driven agent-based simulation perspective.

## The approach: agent-based modelling (ABM)

We employ a computer simulation to model the dynamics of regional knowledge creation in China. Given the overall characteristics of knowledge creation mechanisms, mainly the fact that it is a complex phenomenon driven by different actors and their interactions (see Sect. 4), we find a simulation approach—specifically, agent-based modelling (ABM)—particularly useful. However, it remains fairly underexplored in regional innovation research, with the exception of some recent works (see, e.g. Wang 2018; Sebestyén and Varga 2019; Neuländtner 2020). So far, recent contributions to studying innovation, technological change, and knowledge dynamics in innovation networks via simulation modelling are often implemented at a theoretical and conceptual level (Quintero Ramírez et al. 2017, Vermeulen and Pyka 2016, Maggioni and Roncari 2009, Dawid 2006, Gilbert et al. 2001). Although theoretical models are valuable for theory-building, only recently have a few empirical models aimed at analysing real-world scenarios. Most significantly, Wang et al. (2018) use an ABM to study the diffusion of technologies across Chinese regions, while Neuländtner (2020) studies knowledge creation across European regions. Other studies either have limited geographic or sectoral scope (e.g. Paier et al. 2017) or neglect the complexity of regional innovation systems focusing on aspects of network formation (e.g. Sebestyén and Varga 2019), knowledge diffusion (e.g. März et al. 2006) or learning and innovation dynamics (Quintero Ramírez et al. 2017; Maggioni and Roncari 2009).

ABM is a computer simulation technique widely used to model complex systems. Whereas traditional computer simulation approaches such as discrete event simulation, object-oriented simulation and dynamic microsimulation are generally used to simulate such systems in the fields of engineering, physics and technology, ABM is widely used in the context of social sciences. It differs from other kinds of computer-based simulation in that the simulated entities are modelled and implemented in terms of agents and their respective behaviours (e.g. Conte et al. 1998). The core idea of the ABM approach is to capture emergent macro phenomena generated by the interactions between autonomous, individual, and heterogeneous agents, i.e. from a bottom-up perspective (Schillo et al. 2000). With the simulation model we are able to conduct controlled simulation scenario experiments to replicate real-world concepts, actions, relations, or mechanisms to anticipate future developments and outcomes, that otherwise cannot be predicted by the combination of individual-level interactions (Nikolic et al. 2013).

While the ABM approach can overcome some limitations of more traditional methods commonly used—such as assumptions of linearity, homogeneity, and stationarity—it also faces frequent criticisms. These include (i) a certain degree of ‘ad hocerism’, (ii) its ‘black-box’ character, (iii) the lack of validation using empirical data, and (iv) the inability of agents to respond to policies (see Napolitano 2018 for detailed discussion on the issues). To address these points, we have taken several careful steps. First, we thoroughly selected behavioural rules and mechanisms of interaction based on state-of-the art research on (regional) knowledge. Second, we prioritise transparency by rigorously illustrating and describing the model's components and testing its dynamics and causal mechanisms through sensitivity analyses. Third, we grounded the model strongly in empirical data throughout the whole process of the model development and use (initialisation, calibration and validation). Finally, we tailored the model to the processes of regional knowledge creation featuring a few, but sophisticated learning and adaption mechanisms that are well prone to react to externally induced policy changes and at the same time unlikely to face constraints in their adaption processes.^2^

In general, an ABM follows a structured procedure consisting of initialisation, agent interactions, and outcome evaluation. First, agents are created with defined attributes, behaviours, and decision-making rules that are derived from theoretical assumptions and, in the case of empirical ABMs, also informed by empirical data. These agents operate within an environment that provides contextual constraints and interaction opportunities. Next, agents follow predefined rules to make decisions, adapt to changes, and interact with other agents and the environment, leading to emergent dynamics at agent, but also system level. Over multiple simulation steps, agents update their states based on interactions and learning processes. Finally, the simulation results are analysed by comparing model outputs to real-world data or theoretical expectations, involving calibration and validation to increase model accuracy. This iterative process allows researchers to explore complex system behaviours, test hypotheses, and analyse policy implications.

Before the final model use and application, the model needs to be calibrated and validated. Whereas the calibration process aims at finding values for the input parameters that make the model reproduce patterns observed in reality sufficiently well, the model validation assesses the accuracy of the model’s representation of the real world, e.g. by means of expert evaluation.

## Theoretical model overview

The model’s conception is largely based on a model of simulating regional knowledge creation initially developed for the European case, as laid out in detail in Neuländtner (2020). Hence, the description in this section is deliberately limited to the most fundamental model elements and processes. For a most accurate representation of already well-studied mechanisms of regional knowledge creation, we closely follow state-of-the-art theoretical and conceptual contributions, as well as empirical findings in the fields of regional science, economic geography, and the geography of innovation literature (see Sect. 2) for the model design.

**Fig. 1:**
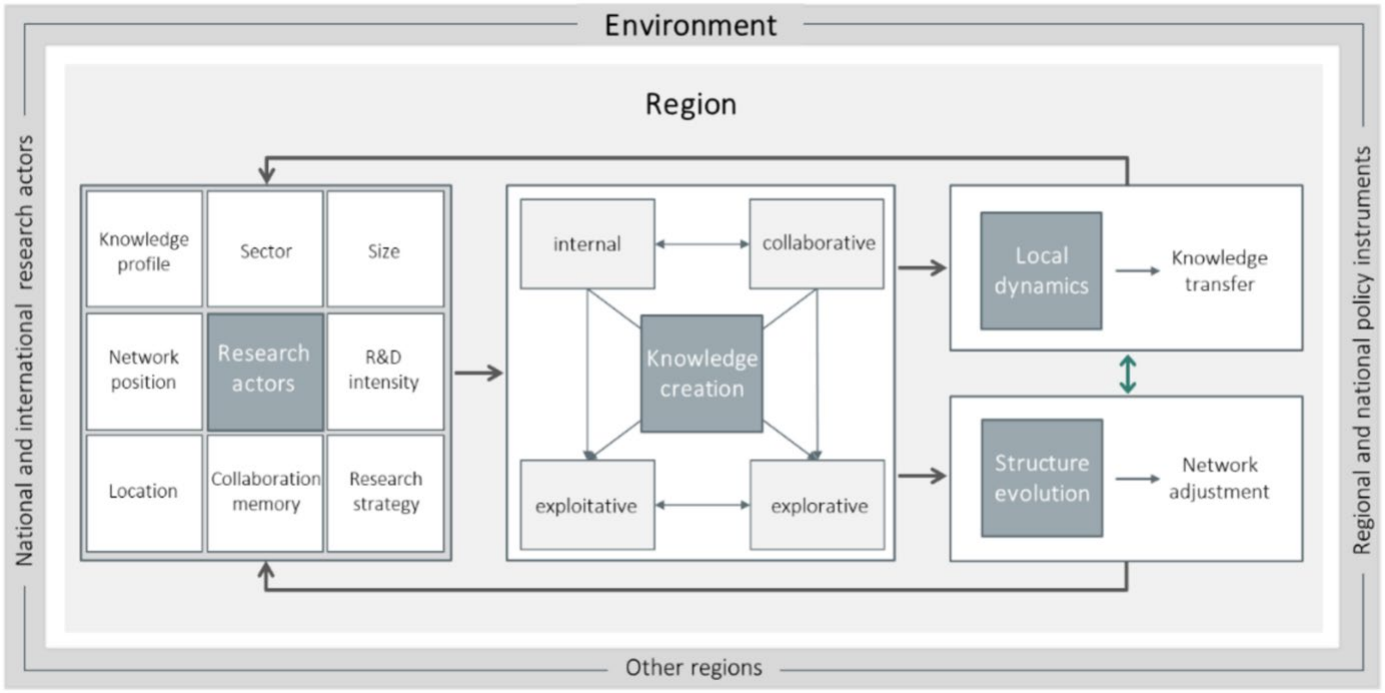
Overview of model elements and processes—region perspective

The agent-based model (ABM) applied in this study follows a structured procedure to simulate regional knowledge creation processes. Initially, the agents representing research actors (firms) are instantiated with empirically derived attributes: knowledge profile (representing the knowledge endowment of the agent, indicative of the technological fields the agent is active in, and the expertise in the respective class), sector (industry sector), size (number of employees), location (Chinese city), and R&D intensity.^3^ Next to these empirically derived attributes, three internal attributes—not derived from empirical data—are added to the agents’ endowments: research strategy, collaboration memory and network position. Both empirically derived and internal attributes affect the agent´s behaviour in the system (Fig. 1).

A network of technologies—labelled as technology space—provides the framework for the knowledge creation process. It is constructed from patent data using the co-occurrences of IPC technological patent classes on patent documents to determine the technological proximity between the classes (Engelsman and Van Raan 1991). Agents navigate this technology space along research paths determined by their knowledge creation mode. The model distinguishes an exploitative (targeted, commercially driven research) or explorative (open-ended, novel research) mode.

In essence, agents create new knowledge in the ABM through a sequence of decisions (a detailed flow chart illustrating the processes and modelling decisions is presented in Appendix A). *First*, they select a research target based on the principle of technological proximity—reflecting the idea of related variety (Frenken et al. 2007) and relatedness (Boschma et al. 2017)—favouring adjacent technology classes that align with their existing expertise. *Second*, they determine their research strategy, choosing between internal research (conducted within their organisation) or collaborative research (partnering with external agents). In the case of collaboration, agents assess potential partners based on geographic proximity, cognitive distance, and historical collaboration memory. Collaboration decisions are further influenced by probabilities estimated through a Spatial Interaction Model (SIM), incorporating factors such as inter-regional connections and empirical collaboration data.

As agents conduct research, their learning process is evaluated iteratively. Research success is assessed using a composite indicator that considers agent-specific characteristics (expertise, R&D capacity, network position) and the technological proximity between current and target technology classes. If successful, the agent updates its expertise and progresses along its research path. If unsuccessful, it may adjust its trajectory or seek alternative partners.

The actual knowledge gained from the learning process is influenced by absorptive capacity, balancing the novelty of new knowledge and its understandability following an inverted U-shape, where both too little and too much overlap in knowledge lead to low gains, indicating an optimal cognitive distance for knowledge sharing (Cowan and Jonard 2004; Nooteboom 1999; Cohen and Levinthal 1990). Additionally, knowledge transfer in collaborative research depends on their cognitive distance (the difference in knowledge between them).

An important element of each ABM is the environment defining the space in which agents operate and providing external influences on decision-making. In the context of regional innovation systems, we see national and international research actors and other regions as external elements of the specific region. Moreover, national and European policy interventions may also affect the city-specific knowledge creation processes, potentially strongly interrelating with agent-specific capabilities. In an ABM, this fact is reflected in the relationship between agent behaviour and its environment comprising external factors—steered by the modeller by means of exogenous parameters.

## Towards an empirical model

The simulation model investigates inter-regional knowledge creation across Chinese cities. We adopt an empirically driven ABM approach, utilising large-scale data sets on regional knowledge creation and research collaboration activities. The empirical foundation is one of the crucial aspects in which the proposed simulation model differs from purely theoretical and conceptual models of regional knowledge creation. A thorough empirical foundation is essential for representing real-world processes, practical applications, and policy analyses since it increases their integrative strength and liability.

We mainly rely on two primary data sources, patent data on the city level drawn from the *Incopat*^4^ database (for initialisation period: 2013–2016 and for calibration period: 2017–2019) and city-level general statistics on, e.g. economic performance, human resources, and the firm and higher-education sector, collected from the China city statistical yearbook (period 2013–2016). In particular, in this model, three central elements are driven by empirical data: model initialisation, calibration of model parameters, and output evaluation. In addition, throughout the model, agents’ decision-making processes are empirically driven by means of statistical figures.

### Model initialisation

At the system level, a crucial aspect is the empirical initialisation of the technology space,^5^ which structures agents' opportunities for knowledge acquisition and guides their learning trajectories. The network of technology classes—comprising the technology space—is constructed using the patent data as extracted from the *Incopat* database for Chinese cities from 2013 to 2016 by determining the co-occurrences of International Patent Classification (IPC) patent subclasses (3-digit) on patent documents. Technological proximities—indicated by the Jaccard coefficient (Rip and Courtial 1984)—between the technology classes indicate the ease of transition between technology fields and the extent of knowledge transfer during the learning process. Agent specification represents another key element of the model’s initialisation. Given the absence of comprehensive micro-level organisational data, agents are generated based on city-level statistics to reflect regional characteristics (using spatial microsimulation).

Since this model's overall aim is to simulate knowledge creation, a particular emphasis is put on the initialisation of the agents’ knowledge profiles. They are constructed by assigning an unique set of IPC patent subclasses based on the patenting activity of each agent’s city’s patent profile, using the Incopat database. In total, the model comprises over 42,000 agents, which is approximately a fraction of 10,000 of the actual number of firms located in the Chinese cities of interest (based on the China city statistical yearbook). These agents are active across 37 industry sectors (2-digit, ISIC) and distributed among 289 Chinese cities (285 prefectures and four municipalities).^6^

### Calibration of model parameters

The calibration process aims to identify parameter values that enable the model to replicate observed empirical patterns with sufficient accuracy (Thiele et al. 2014). To efficiently explore the parameter space while limiting the number of simulations required, Latin hypercube sampling is employed. This stratified sampling method ensures uniform coverage of the entire parameter space, thereby selecting a representative subset of scenarios while minimising computational demands.

The core of the empirical calibration lies in fitting of model parameters so that the resulting output variables align with selected empirical measures. Following Thiele et al. (2014) two different strategies for fitting model parameters can be distinguished: (i) best-fit calibration, which seeks the parameter set that minimises the deviations from empirical observations and (ii) categorical calibration, which defines acceptable ranges for each calibration criterion. For this model, we follow a hybrid approach by transforming the categorical criteria into a best-fit criterion. This is operationalised through conditional equations and the specification of a cost function, which penalises deviations from acceptable empirical ranges (which are defined externally).

1 $${\text{criterium}}_{r} \left( {x_{r} } \right) = \left\{ {\begin{array}{*{20}l} 0 \hfill & {{\text{if}}\,\,\,x_{\min } \le x_{r} \le x_{\max } } \hfill \\ {\left( {\frac{{{\text{mean}}\left( {x_{\min } ,x_{\max } } \right) - x_{r} }}{{{\text{mean}}\left( {x_{\min } ,x_{\max } } \right)}}} \right)} \hfill & {{\text{else}}} \hfill \\ \end{array} } \right\}$$

2 $$\cos t\left( {x_{r} } \right) = \mathop \sum \limits_{r = 1}^{R} {\text{criterium}}_{r} \left( {x_{r} } \right),\,\,\,r = 1, \ldots ,R$$

where $x_{r}$ are corresponding simulation results and $R$ is the number of calibration criteria included. For each selected empirical measure, an acceptable value range is defined. If the simulated value lies within this interval, no costs are incurred; otherwise, a cost factor based on the squared relative deviation to the mean value of the acceptable range is assigned. The final cost function is the sum of the individual costs of each criterion. Finally, the parameter combination associated with the lowest overall cost is chosen as the one that best fits the real-world system. Applying this cost function approach enables combining multiple calibration criteria into one single decision criterion (Thiele et al. 2014).

Empirically, four measures are chosen as criteria for the cost function: (i) the total number of patents in the agent population, (ii) the patenting profile across regions, (iii) the patenting profile across patent classes, and (iv) the region’s degree centrality in the collaboration network.^7^ In choosing these criteria, we reflect quantity- (absolute knowledge output) and quality-related (regional, technological variety and number of network connections) dimensions of regional knowledge produced by agents. The empirical reference dataset for all measures is the patent performance of Chinese cities. The empirical measures are calculated as aggregate values over the years 2017 to 2019; the calibration is performed with the simulated patent output after 40 time steps (see Appendix A for calibrated system parameters).

The emergence of a patent from an instance of knowledge gain is determined through an independent evaluation mechanism, implemented as an empirical output filter. This filter is based on econometrically estimated coefficients that influence the patenting propensity of an individual agent by means of a region-specific probability that is determined by regional characteristics (Gross Regional Product (GRP) per capita, number of students per inhabitants, number of firms, and degree centrality).^8^ In matching the simulated patent output to the empirical data, we establish a meaningful correspondence between the model’s time steps and real-world time.^9^ The calibrated parameter set defines the baseline scenario, which is used as a reference scenario for the simulations presented in Sect. 4.

**Fig. 2:**
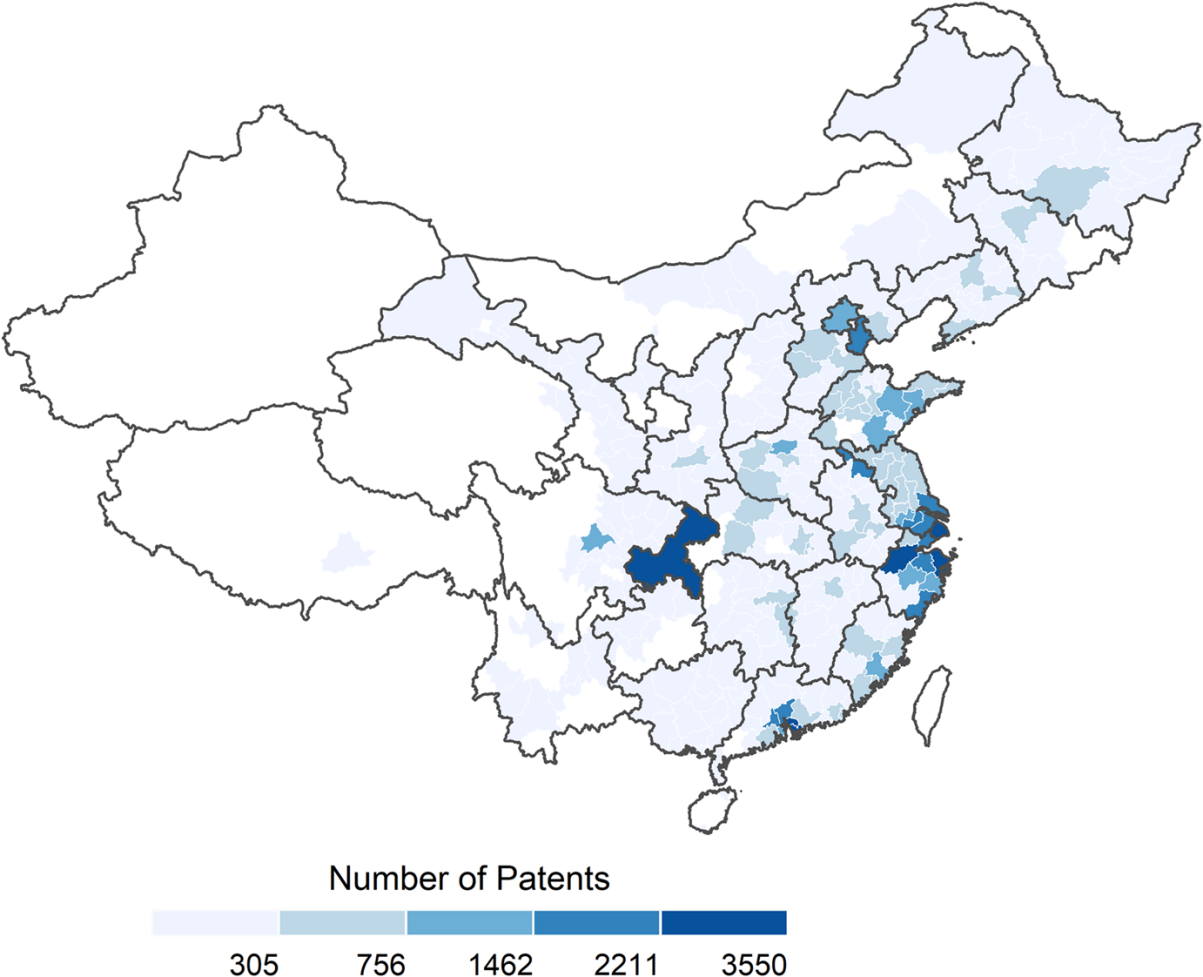
Distribution of simulated patents in calibrated baseline scenario

Figure 2 shows the distribution of simulated patents for the calibrated baseline scenario after 120 time steps (representing three years). 10 Looking at the city level, it shows the metropolitan area of Shanghai with the highest patent intensity, interestingly followed by Ningbo, Chongqing and Dongguan, all producing a larger number of patents in the model than empirically observed (see Appendix A). This may be related to the large number of firms located in these cities, playing an important role in the knowledge creation process of the model. Although some larger cities are overrepresented regarding their simulated patent counts, the empirical distribution of cities’ patents is much more skewed than the simulated counts. Nevertheless, the spatial distribution of patents in the calibrated model correlates highly with what is empirically observed, which is a strong indication for the representativity of the agents’ behaviour on the city level.

## Exploring dynamics of increased collaboration intensity

In this section, we focus on different modelling scenarios to infer the main research question: how may inter-regional R&D collaborations affect regional knowledge creation processes under specific conditions? Specifically, we illustrate how increased inter-city networking affects knowledge creation, both from a quantitative perspective on knowledge outputs at the system and city-specific level, and from a qualitative perspective regarding changing technological profiles related to increased collaboration intensity. From the simulation model’s perspective, these aspects are implemented by changes in the parameter settings. In particular, the collaboration probability parameter is increased by 50 percentage points. By this, we represent changes in the agents’ collaboration behaviour resulting in increased inter-city R&D collaboration network density and changes compared to the calibrated model. In what follows, we initially take a perspective on cities (collectively and individually) to discuss the influences of increased collaboration (Sect. 6.1) before we put emphasis on the technological dynamics (Sect. 6.2).

### Cities’ perspective

Reviewing the results of the increased collaboration scenario reveals some novel findings, not only in a scientific context underlining some of the theoretical assumptions discussed in the literature but also from a policy perspective, pointing to some interesting policy implications, in particular in terms of more thorough measures to increase the R&D collaboration propensity across Chinese cities. Before we look into the results along certain dimensions, Table 1 provides an overview of some overall summary statistics on knowledge outputs and changes in regional concentration. Note that the increased collaboration scenario is based on 120 timesteps (3 years), reporting averages of three model runs.^11^ First, it can be seen clearly that increased collaboration overall increases the intensity of knowledge production at the system level (China as a whole). After 120 time steps, the increased collaboration scenario produces over 110 k patents, in comparison with about 100 k patents in the baseline scenario over the same time period, i.e. an increase of 10% in this comparably short time frame. On average, most cities show this increase in patents, reflected by the higher mean but also median of patents produced in the increased collaboration scenario. However, it seems that the benefits of collaboration markedly differ across cities. While, on average, all cities benefit from increased collaboration, there are strong differences in relative gains. Some cities seem to benefit enormously, leading to a higher concentration, although, on average, most cities benefit.

**Table 1:** Baseline versus increased collaboration scenario: basic summary statistics

	Baseline scenario	Increased collaboration scenario
Total number of patents^*^	100,359	110,666
Mean number of patents^*^	362	400
Median of patents^*^	158	165
Skewness of patents^*^	3.35	3.5
Kurtosis of patents^*^	12.11	13.53
Regional concentration^**^	0.013	0.015

Increased collaboration scenario based on 120 time steps; averages of three simulation runs; * across 289 Chinese cities; ** Herfindahl concentration index

**Fig. 3:**
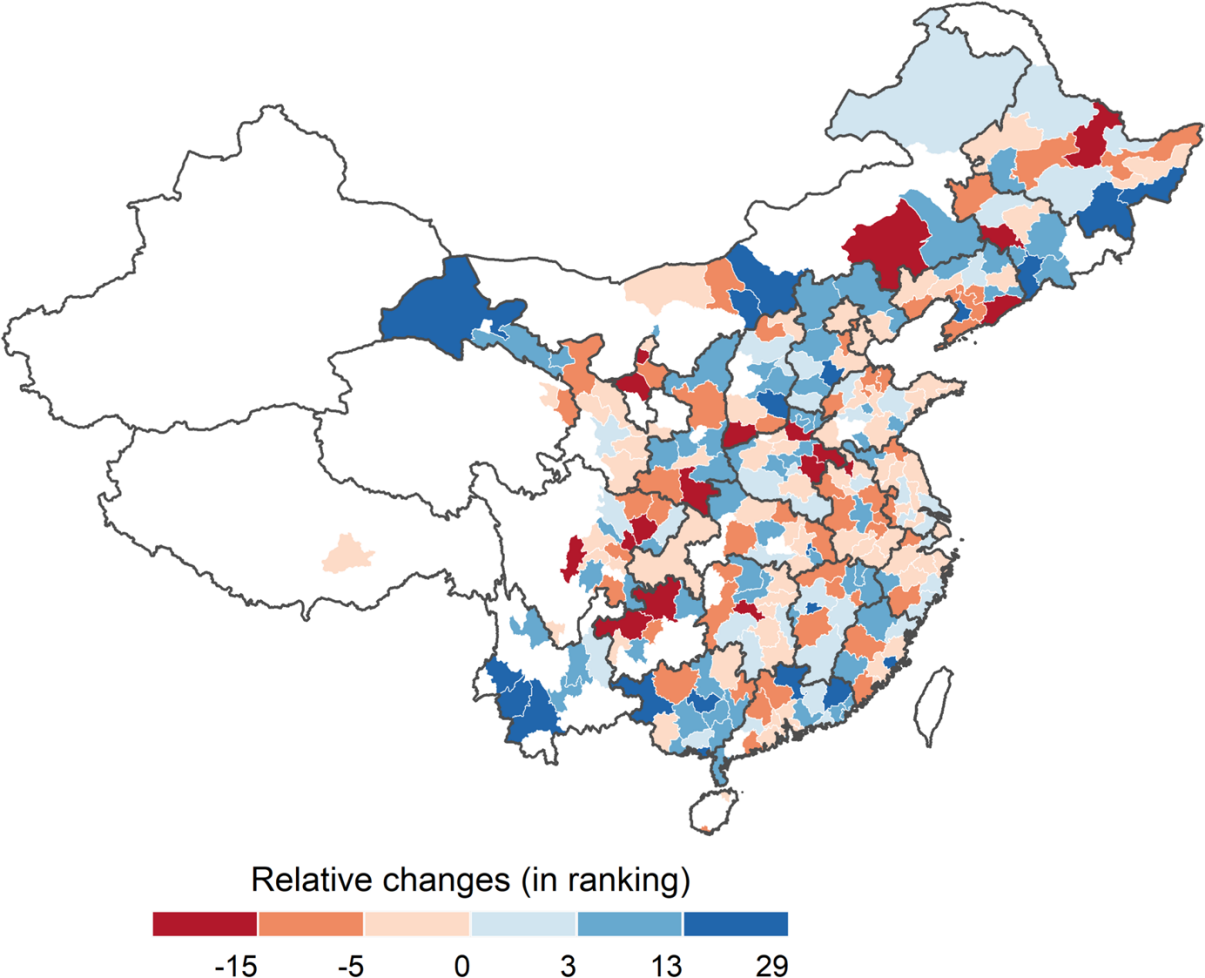
Cities’ changes in the ranking of patenting

This interesting pattern leads us to dig deeper into detailed changes at the level of individual cities. Initially, Fig. 3 shows the changes in the cities’ patent ranking (i.e. relative changes^12^); here, cities are ranked by number of patents (cumulated after 120 time steps) in the baseline scenario and the scenario of increased collaborative research, while differences between the two rankings are illustrated in the map, positive values represent a rise in rank (better ranking) due to increased collaboration; negative values suggest a loss in rank due to increased collaboration. It can be seen that cities with the highest rises in rank include^13^ Changzhi, Yingkou, Beihai, Lincang, Shaoguan, Hohhot, Mudanjiang, Laibin, Putian, Baise, Xinyu and Hengshui, while cities with highest losses in rank include Bijie, Suining, Yuncheng, Chifeng, Yichun, Zhoukou, Xinxiang, Zunyi, Loudi, Siping and Ya’an. Obviously, cities with an insufficient endowment of basic knowledge resources (e.g. human resources) are not able to lever additional knowledge outputs from collaboration; in relative terms, they even decline. In contrast, cities with some basic endowments available can better exploit their potential for knowledge creation through an increased collaboration intensity.

Moreover, traditional knowledge hubs (i.e. Shanghai, Shenzhen, Beijing, Guangzhou) do not climb up in the ranking since most of them are on the top already, but also for the number of additional patents produced we just observe—if any—rather low gains in the increased collaboration scenario for these core cities. This is in line with earlier results for the European case, showing that relative gains from R&D collaboration networks are much higher for lagging than for core regions (see Neuländtner and Scherngell 2022, Wanzenböck et al. 2020).

**Fig. 4:**
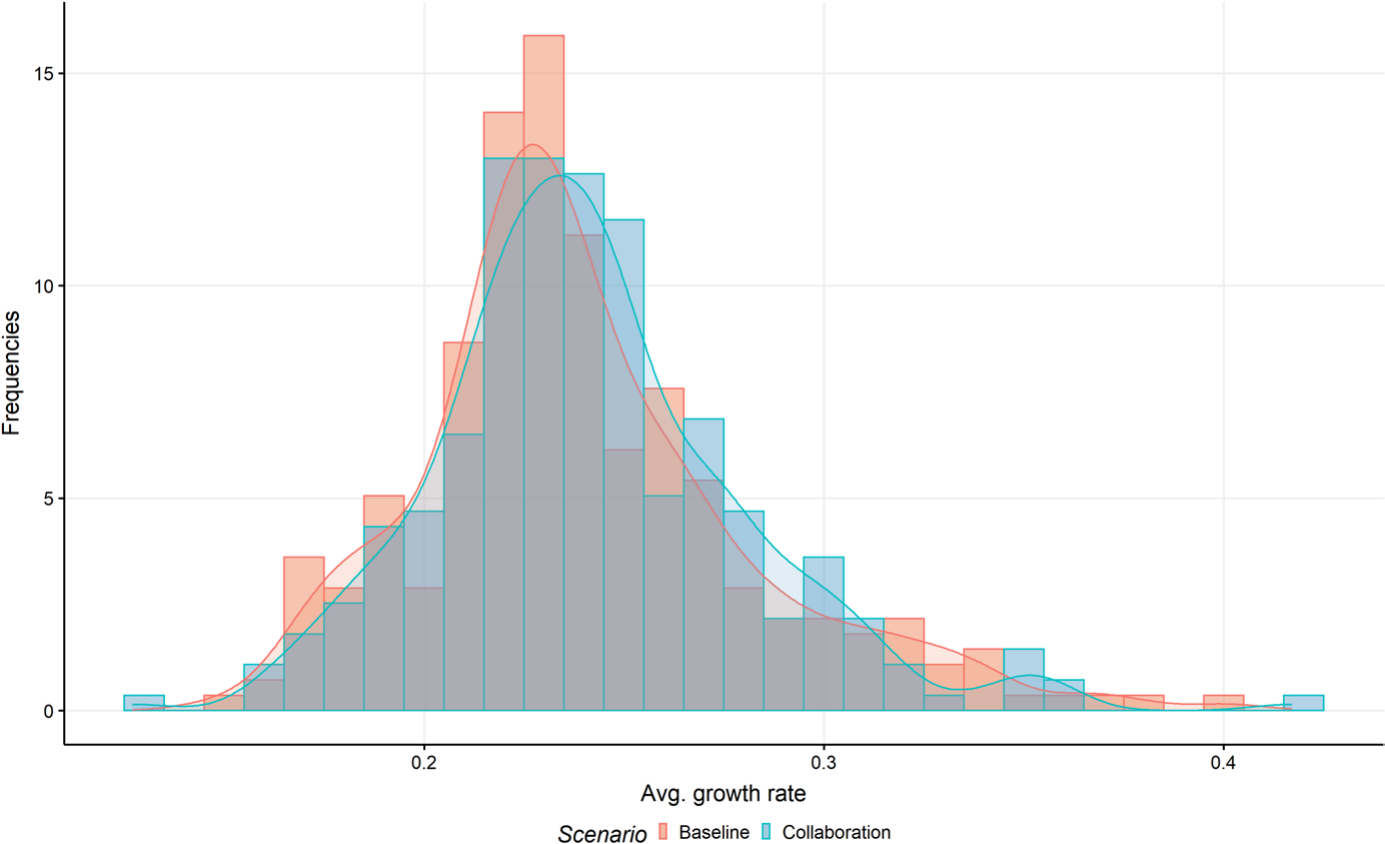
Differences in cities’ average patent growth

To complement this analysis, Fig. 4 underlines the difference in the distributions of the cities’ average (yearly) patent growth rates over the observed period. For each simulated 12 time steps (i.e. approx. one quarter of a year), the patent growth rate is calculated; the average of these growth rates for each city is displayed in the histogram (with densities). We can find a slightly shifted distribution to the right of the average growth rates for the scenario of increased collaboration intensity (compared to the calibrated baseline scenario), confirming the generally higher average growth rates, as described above already. However, the distribution of the growth rates is less concentrated in the case of an increased level of collaborative research (also, the median value in the collaborative scenario is smaller than in the baseline scenario). Again, it can be seen that medium, slightly above average cities benefit most from increased collaboration. Regions with a growth rate of about 0.25% in patenting nearly double their numbers in the increased collaboration scenario, while regions with low growth rates in the baseline scenario (below 0.2%) even show lower patenting frequencies in the increased collaboration scenario than in the baseline scenario.

**Fig. 5:**
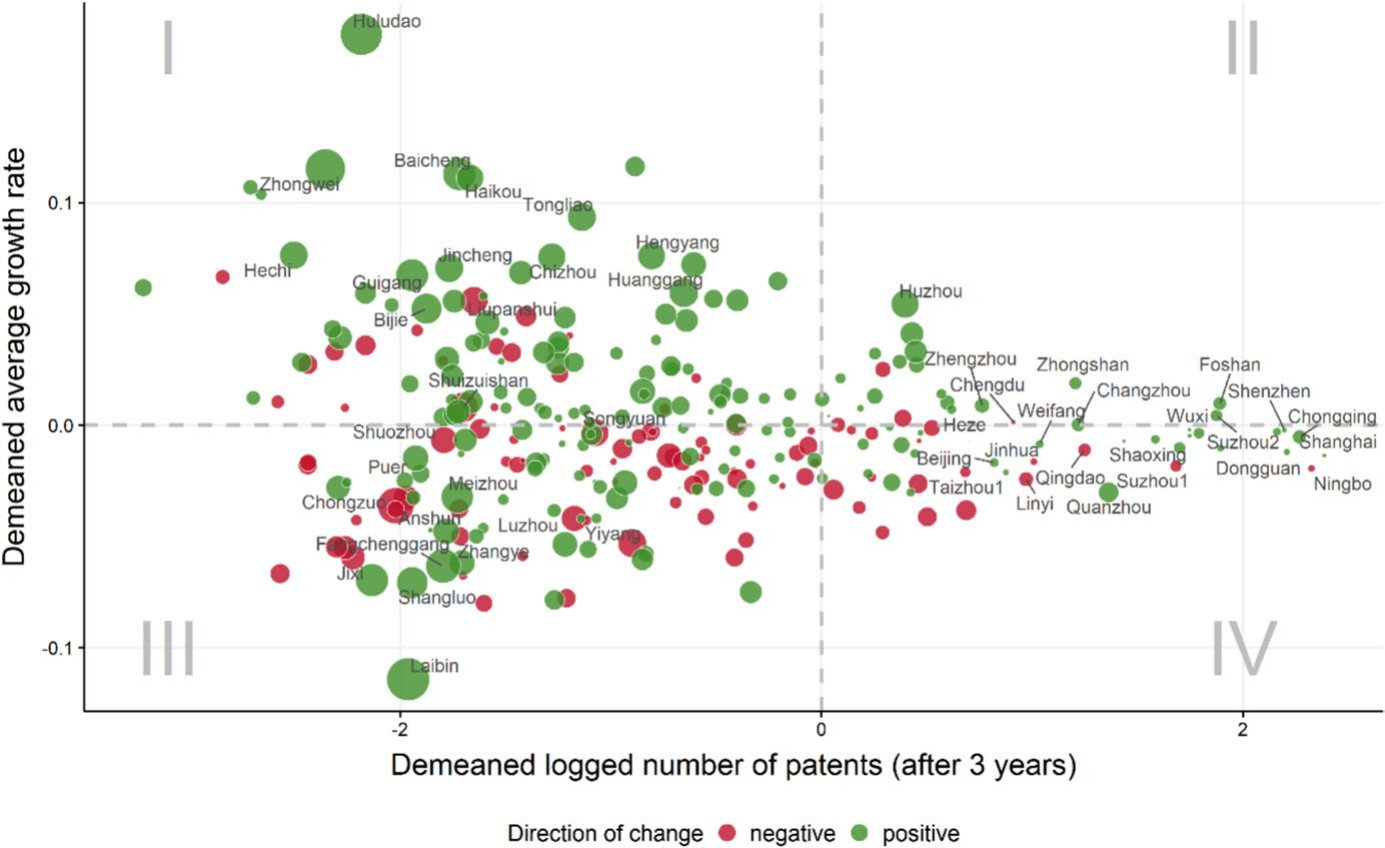
Portfolio of cities’ changes in patenting and patent growth

To disentangle ‘winners’ and ‘losers’ in a more insightful way, Fig. 5 shows two angles on the scenario of increased collaboration^14^: *First*, the figure illustrates a portfolio that allows to benchmark cities in terms of their patenting activity and/or patent growth with increased collaboration; represented by the cities’ location (discs) in the four quadrants. *Second*, the figure displays benefiting and losing cities in terms of their changes in patent activity and patent growth in the scenario of increased collaboration (compared to the baseline scenario); represented by the colour and size of the discs.

Focusing on the first angle, we distinguish four cases (quadrants): cities with a small number of patents and low patent growth, cities with either a small/high number of patents and/or a high/low patent growth, and cities with both, a high patenting activity and a high patent growth—in the simulated collaboration scenario. As also observed for the baseline scenario, the number of patents is highly skewed in the scenario of increased collaboration. Moreover, the cities’ patent growth rates are more scattered around the mean growth rate, with lower patent counts. This indicates a few already quite mature and well-established cities in terms of their innovation capacities (e.g. Chongqing, Shenzhen, Shanghai), while cities with lower patent activities show a high amplitude of patent growth, pointing out cities with a high potential to catch up on leading cities (quadrant I, e.g. Nanchang and Zhoushan) or potentially stagnating or declining cities (quadrant III, e.g. Keifeng and Huangshi)—in terms of their patent activity. Additionally, in this respect, a simple regression analysis with the patent’s initial level and growth rate confirms slight convergence tendencies for both, the baseline and the scenario of increased collaboration.

The second angle highlights which cities benefit from (increased) collaboration. While the discs’ colour signals the ‘direction’ of the change—discs in dark green show positive changes, indicating higher patent counts and/or a higher patenting growth rate for these cities in the scenario of a generally increased collaboration probability—the discs’ size represents the ‘strength’ of this change. Overall, the positive changes of increased collaboration prevail in their strength (dark green discs have a generally larger disc size), implying a positive effect of collaborative R&D on the system level. On the city level, positive changes entail a city’s increased patent output and/or patent growth resulting from additional R&D collaborations.

An additional interesting perspective (deducted from analyses underlying Fig. 5 but not directly visible therein) is to highlight cities that are able to increase their performance (w.r.t. the number of patents and patent’s growth rate), once collaboration is intensified, by changing from one to another quadrant; specifically, transitions from quadrant III to I (‘from losers to emerging/rising stars’), from quadrant III to II (‘from losers to stars’), and from quadrant I to II (‘from emerging/rising stars to stars’). Moreover, we are interested in those cities that show high numbers of (demeaned) patents in quadrant III but with negative development—these cities are not capable of leveraging their patenting capabilities with increased collaboration.

We find that cities with transitions from quadrant III to I (‘from losers to emerging/rising stars’) are manifold. The ones with the biggest changes involve the cities of Fushun, Handan, Bozhou, Zhoushan, Qujing, Chizhou, Tongliao, Baoji, Hulunbuir, Guigang. There is only one city with a transition from quadrant III to II (‘from losers to stars’), namely the city of Shantou. Actually, it is worth mentioning that in 2022, Shantou was approved by the Chinese Ministry of Science and Technology to become a pilot of the so-called National Innovation City Pilot Policy based on its excellent innovation capacity.^15^ Transitions from quadrant I to II (‘from emerging/rising stars to stars’) do not occur, while cities that show high values of (demeaned) number of patents in quadrant III but with negative development involve cities like Xian, Jiaozuo, Jiujiang, Chuzhou, Zaozhuang, Ganzhou, Xiaogan, Jingzhou, Nanning, Mianyang, Anshan. While these results are rather descriptive in terms of the individual cities, it can be extremely informative by digging deeper into identifying the reasons for these increases or declines which could be an important element of future research in this direction. This may produce important policy conclusions at the individual city level, probably pointing—for ‘losers’—to specific alternative measures that may be initiated to be able to gain from collaboration more purposefully.

### Technologies’ perspective

While the results derived at the level of cities are interesting and relevant per se, they become more significant in a policy context when complementing them with a perspective on technological dynamics driven by increased collaboration activities. Such technological dynamics can be observed at the systems level (in our case China as a whole), discussing how the knowledge production intensity in certain technologies changes in a scenario of increased collaboration. This can be very well related to the discussion of technological heterogeneities and different epistemological regimes at stake in creating new knowledge (see Neuländtner and Scherngell 2022). Moreover, employing a combined technology-city perspective enables much more detailed insights into how the technological profiles of a specific city may develop in an increased collaboration scenario, providing important pointers to regional policymakers at a very detailed level of granularity.

**Fig. 6:**
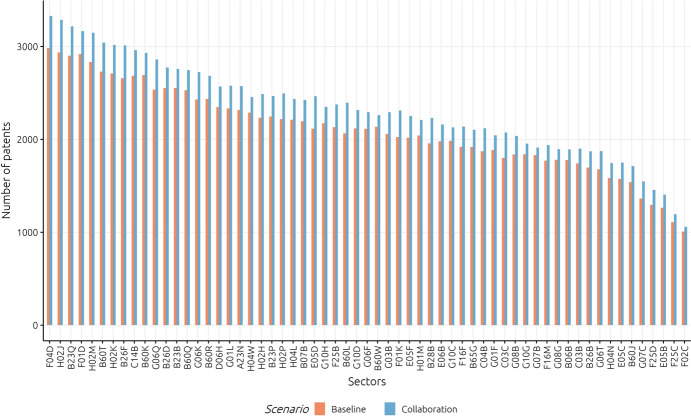
Chinese patent profile (IPC classes with more than 1000 patents)

Starting with a system-level perspective, we initially reflect on the technological dynamics of China as a whole in a scenario of increased collaboration. Figure 6 illustrates the overall technological profile of China in terms of its distribution of patents across selected IPC classes—for the baseline and the increased collaborative scenario.

The IPC classes are sorted by the number of patents in the baseline scenario (meaning of IPC classes is given in Appendix C), with only IPC classes with more than 1000 simulated patents (cumulated over 120 steps being displayed. It can be seen that most important technological fields come from F-, B- and H- (three of each of them among the top 10), in particular, H02 classes (conversion of electrical power), as well as classes F04D and F01D related to non-positive displacement of machines or engines, and B23 related to machine tools. High intensity is also shown for H04 classes related to digital communication. Overall, in the scenario of increased collaboration intensity, patent counts are higher for each IPC class displayed—which corresponds to the generally higher number of patents in this scenario, i.e. at the system-level differences in the positive effect of increased collaboration on knowledge outputs in different technologies are relatively minor.

However, this is not necessarily the case when taking a look at the simulated city-specific patent portfolios (such as, e.g. for Beijing, Shanghai, Shenzen and Chongqing; see respective figures in Appendix B) that reveal different technological dynamics in different regions under the increased collaboration scenario. For instance, for the city of Beijing, we can observe that the technologies B06B (*Generating or transmitting mechanical vibrations*), C14B (*Mechanical treatment or processing of skins, hides, or leather in general*), or H02M (*Conversion of electric power*) show a lower knowledge output in the increased collaboration scenario, while other technologies (in particular F01D and F04D on *non-positive displacement machines or engines*) gain enormously, nearly increasing their patent output by about 25%. Accordingly, whether benefits from collaboration can be realised is not only technology- but even more, region-specific, depending on the place-based factors locally moderating collaboration benefits in specific technologies differently.

**Fig. 7:**
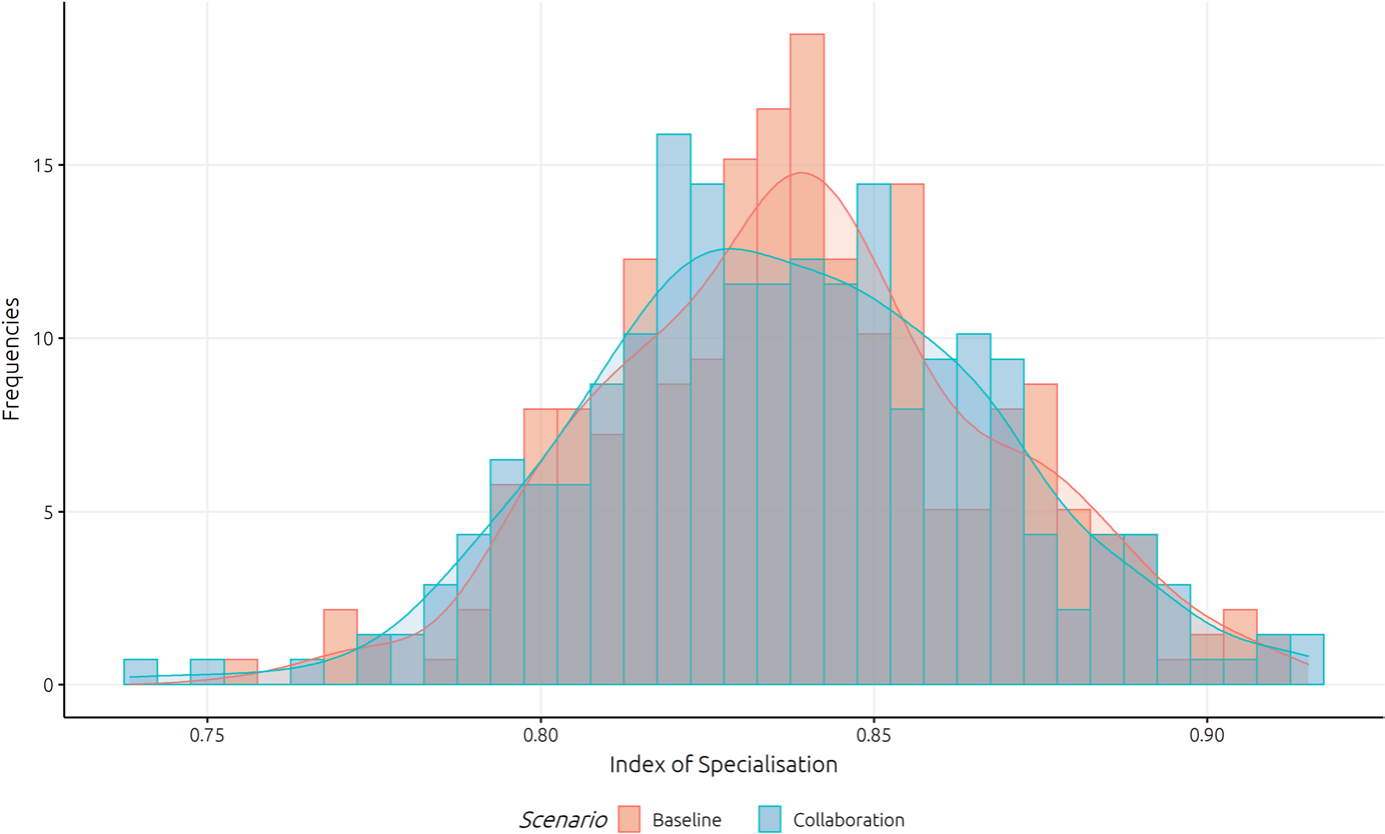
Differences in the distribution of cities’ technological specialisation

Figure 7 underlines the previous findings, showing the differences in the distribution of cities’ technological specialisation comparing the baseline and the increased collaboration scenario.^16^ Whereas, the distribution of the baseline scenario is much more pointed with a higher mean value, the distribution of the scenario with increased collaborative research is less concentrated (and with a lower mean and median value).

This indicates that a higher collaboration intensity results in a less concentrated technological specialisation; technological specialisation tends to be more equal across cities when they engage more in collaboration (the mean index for specialisation decreases from the baseline to the increased collaboration scenario from 0.839 to 0.837). This underlines assumptions from STI policy concepts, that collaboration may be one of the most important vehicles for regions to diversify into related technologies.

## Concluding remarks

R&D collaboration networks are increasingly viewed as ‘carriers’ of knowledge, enabling easier and more efficient access to new specific pieces of knowledge or capabilities often located further away in geographical space. While research debates have long viewed regions as central places where knowledge creation is based and shaped—due to the importance of geographically sticky, tacit knowledge for innovation—more recently, the crucial role of the openness of such local systems to cross-regional interactions has been stressed. Here, the global embeddedness of regions in networks of researching and innovating actors located further away in geographical space is assumed to be a crucial impetus for knowledge creation at the regional level and is therefore also increasingly researched in recent empirical works due to new empirical and methodological opportunities for tracing R&D collaboration networks in geographical and technological space (see Scherngell 2013 for an overview).

This study addresses two main research gaps in the literature stream investigating the role of R&D collaboration for regional knowledge creation. First, there is only scarce research in this direction at a systematic and empirically comprehensive level for China, in particular at detailed regional breakdowns below province level. Second, empirical works so far are done at aggregate levels of countries or regions, missing a deeper understanding of the underlying micro-dynamics that play a role in shaping regional knowledge creation capabilities. The first gap is addressed by a detailed collection of data on R&I activities and outputs of 289 Chinese cities, while the second gap is approached by developing an empirically calibrated ABM. The objective has been to simulate the dynamic relationships between regional knowledge creation and inter-city R&D collaboration in China, identifying respective impact scenarios of increased collaboration along different main aspects, including knowledge outputs as captured by patents at the system and city level, different regional development paths under conditions of increased collaboration, as well as expected technological dynamics.

The results are promising and interesting in several dimensions, both in a scientific as well as an innovation policy context, but also in methodological terms. *First*, we illustrate that the ABM perspective—when empirically calibrated in a very detailed way like accomplished in this study—is not only able to re-create the actual status of regional knowledge creation in China based on organisational-level behaviours (bottom-up), but also to illustrate quite elegantly different nonlinear, and non-stationary mechanisms at stake and how specific driving forces, here increased R&D collaboration, transform these mechanisms in different aspects. This shows the great potential of the model, not only to focus on the emergent aspects of R&D collaboration, but also on other relevant and interesting drivers of knowledge creation and innovation not addressed in this study. *Second*, looking at the general role of R&D collaboration, we show that increased collaboration intensity produces markedly higher knowledge gains at the system level (China as a whole). Moreover, the patent growth rate is overall increasing and more de-concentrated spatially, pointing to a slight convergence. *Third*, benefits differ across cities, i.e. local conditions are extremely important in moderating whether a city can increase knowledge outputs when R&D collaboration is increased. In essence, already very established cities (e.g. Shanghai, Guangzhou, Shenzhen) benefit relatively less than follow-up or catching-up regions (e.g. Shantou, Fushun, Handan, Bozhou, Zhoushan, Qujing, Chizhou, Tongliao, Baoji, Hulunbuir, Guigang)—an important argument for policies supporting cohesion across Chinese cities. However, a minimum endowment of specific resources is needed to benefit from collaboration. For regions with too few region-internal endowments (e.g. human resources, number of firms), returns from collaboration cannot be translated into endogenous knowledge gains. *Fourth*, growth in patenting is not very different across different technologies when considered at the system level but very differently shaped at the level of individual cities. Technology-specific knowledge gains from collaboration heavily depend on the place-based factors locally moderating collaboration benefits in specific technologies differently.

These results are not only relevant in progressing empirical research on R&D collaboration and knowledge creation, but even bear important contributions to the theoretical debate, with some assumptions being confirmed at a micro-level (e.g. that increased collaboration leads to higher knowledge outputs), and other new aspects being brought to theory (e.g. technological heterogeneities in knowledge gains from collaboration are highly dependent on moderating local conditions). This paper helps to show a potential change of game regarding the effect of cross-regional R&D collaboration on different cities and the whole country. From a policy perspective, the study results provide some implications for both national Chinese innovation policies, clearly pointing to recommend an even more intensive strategy fostering R&D collaboration across regional boundaries, and in particular also for the first time at detailed regional (city) level, providing potential regional catching-up pathways in light of current local knowledge endowments and technological profiles. Hence, this paper indicates that the increase of inter-city R&D collaboration can narrow down the economic gaps across regions, benefiting the overall national development.

Against this background, some future research ideas come to mind. *First*, clearly, the developed model should be mobilised for other policy scenarios, shifting attention to further driving factors of regional knowledge creation of particular policy interest. The role of agglomeration forces could be one aspect or a stronger focus on important dimensions of future development, such as specific technological domains relevant for green transformation. Another aspect in the policy realm could cover regions’ technological diversification processes based on technological (un) relatedness and their relation to technological change and long-term growth (Boschma et al. 2023; Maggioni et al. 2024). *Second,* the potential of green technologies for technological dynamics at the Chinese city level could be explored in more detail: While China as a whole thrives in green technology development—according to the OECD Green Growth Indicators, Chinese environmental technology patent applications grew by 1040% from 2000 to 2011, as compared with 78% in the OECD—it has been questioned whether the right green technology innovation policy schemes are sufficiently adapted to the regions´ specificities in China. *Third*, further methodological refinements of the model can be envisaged, mainly related to increase computational speed in order to be able to enlarge the time horizon for simulation runs to test results more robustly. Overall, this would increase applicability to other questions but also territories of interest.

## Figures and Tables

**Fig. 8:**
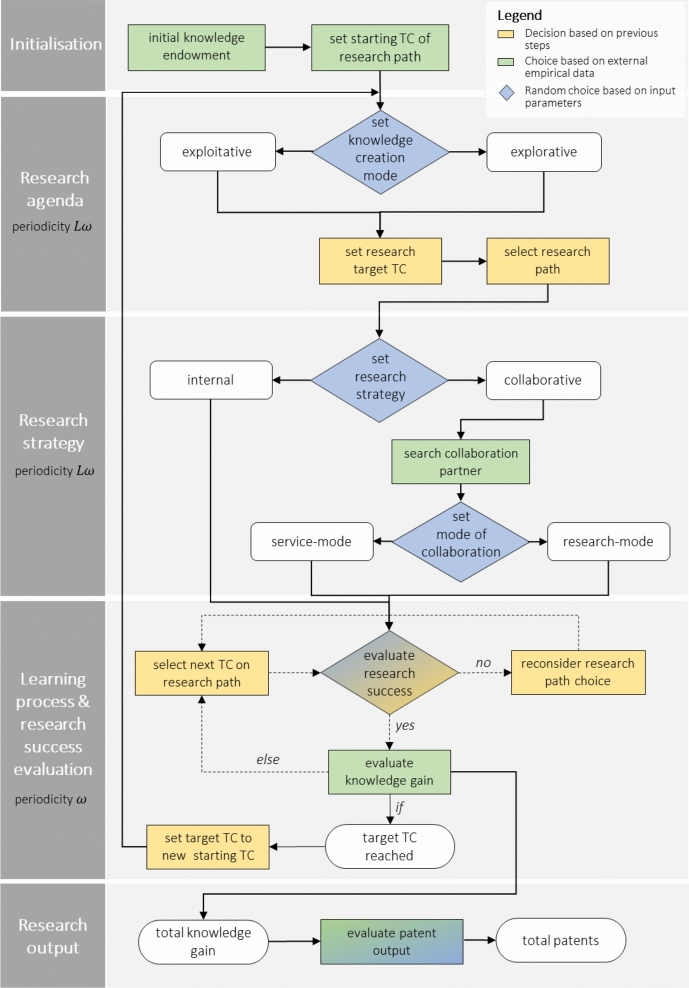
Detailed illustration of agent’s knowledge creation process

**Fig. 9:**
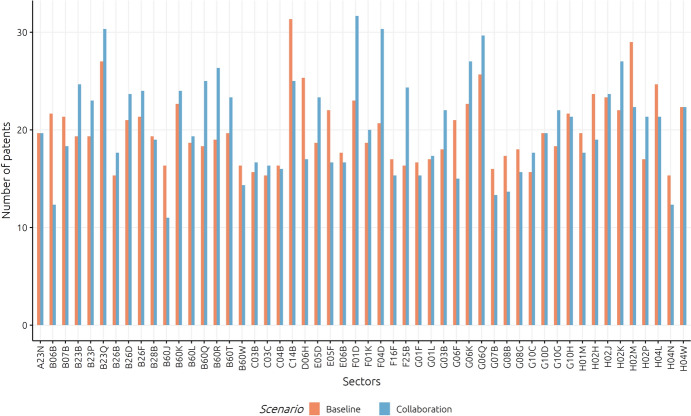
Technological patent profile of Beijing (IPC classes with more than 15 patents)

**Fig. 10:**
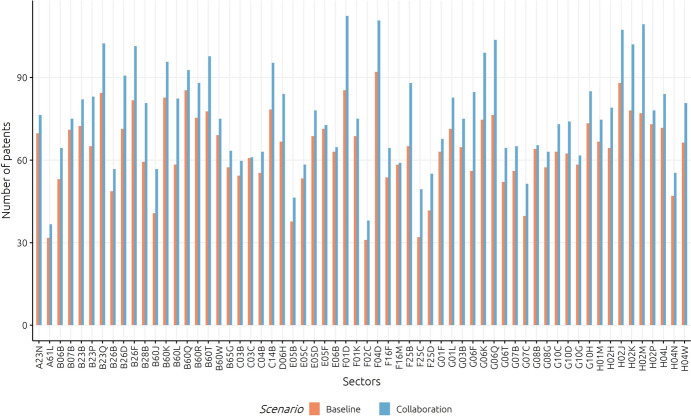
Technological patent profile of Shenzhen (IPC classes with more than 30 patents)

**Fig. 11:**
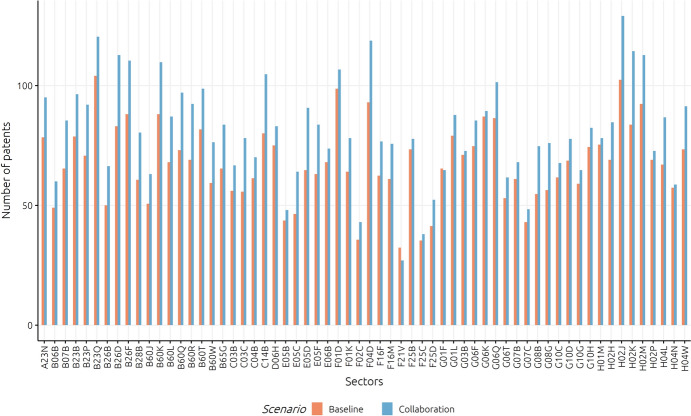
Technological patent profile of Chongqing (IPC classes with more than 30 patents)

**Fig. 12:**
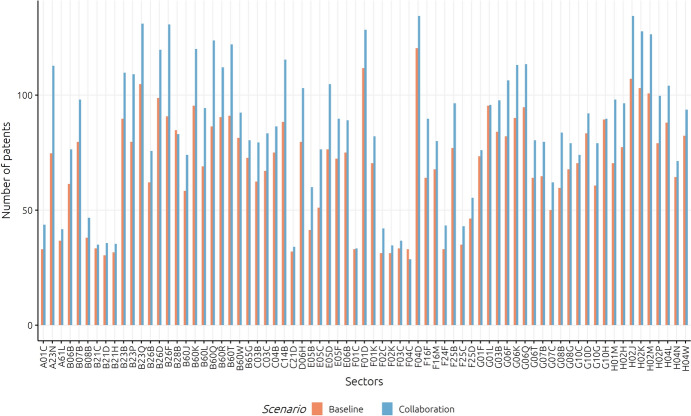
Technological patent profile of Shanghai (IPC classes with more than 30 patents)

## Appendix A

See Tables 2, 3, 4 and Fig. 8.

**Table 2:** List of regions with number of patents (observed, calibrated and increased collaboration; in descending order by observed patents)

City	Region	Population in 10 k (2016)	Empirical patents (avg. 2014–2016)	Calibrated patents (baseline)	Patents (increased collaboration)	Direction of change
Shenzhen	Southcentral	385	5705.3	3001.3	3593.7	+
Beijing	North	1363	3547.7	862.7	907.0	+
Shanghai	East	1450	1711.0	3549.3	4341.0	+
Suzhou1 (Jiangsu)	East	678	1043.7	2085.3	2150.3	+
Chengdu	Southwestern	1399	906.3	969.3	994.0	+
Guangzhou	Southcentral	870	711.3	1780.0	1945.3	+
Hangzhou	East	736	643.3	2806.0	3469.7	+
Qingdao	East	791	621.7	1314.7	1391.0	+
Nanjing	East	663	511.3	649.7	628.0	–
Dongguan	Southcentral	201	492.7	3027.7	3632.3	+
Zhongshan	Southcentral	161	372.0	1256.3	1334.3	+
Foshan	Southcentral	400	357.7	2210.7	2645.7	+
Tianjin	North	1044	347.0	2111.3	2398.0	+
Changzhou	East	375	344.3	1233.3	1350.7	+
Jinan	East	633	332.7	521.0	534.0	+
Ningbo	East	591	296.7	3376.3	4088.0	+
Wuhan	Southcentral	834	288.0	646.3	639.3	–
Chongqing	Southwestern	3392	258.3	3204.7	3856.0	+
Wuxi	East	486	247.7	1997.0	2290.0	+
Quanzhou	East	730	242.7	1324.0	1559.7	+
Xiamen	East	221	218.0	445.0	460.7	+
Xian	North	825	207.3	326.0	331.7	+
Nantong	East	767	196.0	1846.7	2186.7	+
Wenzhou	East	818	193.3	1885.0	2292.7	+
Changsha	Southcentral	696	178.0	749.3	794.7	+
Lianyungang	East	534	171.7	366.3	344.0	–
Shenyang	Northeastern	734	166.0	541.3	588.3	+
Fuzhou1 (Fujian)	East	687	165.3	568.0	579.7	+
Dalian	Northeastern	596	162.7	455.0	437.3	–
Wuhu	East	388	159.0	475.7	481.3	+
Zhengzhou	Southcentral	827	158.7	825.3	857.3	+
Hefei	East	730	154.0	603.7	632.0	+
Huizhou	Southcentral	364	147.7	555.0	625.7	+
Jiaxing	East	352	143.0	2189.7	2651.3	+
Zhuhai	Southcentral	115	138.7	493.3	610.3	+
Luoyang	Southcentral	737	117.7	400.0	423.7	+
Taizhou1 (Zhejiang)	East	508	111.7	844.7	959.0	+
Harbin	Northeastern	962	111.7	316.3	340.7	+
Nanning	Southcentral	752	104.3	208.0	225.3	+
Shaoxing	East	445	102.3	1462.3	1679.0	+
Yantai	East	655	101.3	627.7	614.0	–
Nanchang	East	523	97.3	359.0	325.3	–
Kunming	Southwestern	560	83.3	250.0	279.7	+
Anqing	East	529	82.7	362.7	380.3	+
Zhenjiang	East	272	77.7	628.3	621.7	–
Weifang	East	901	72.3	963.3	1124.7	+
Mianyang	Southwestern	545	72.0	193.3	200.0	+
Shijiazhuang	North	1038	71.3	639.7	675.7	+
Taizhou2 (Jiangsu)	East	600	70.7	747.3	790.3	+
Changchun	Northeastern	753	68.3	381.7	414.3	+
Liuzhou	Southcentral	386	64.3	158.3	172.3	+
Huzhou	East	265	62.7	641.0	594.7	–
Jinhua	East	481	61.3	1010.3	1096.0	+
Guiyang	Southwestern	401	61.0	212.3	196.3	–
Guilin	Southcentral	534	60.3	154.0	151.0	–
Maanshan	East	229	56.3	296.0	265.3	–
Weihai	East	256	55.3	442.0	478.0	+
Xuzhou	East	1041	52.0	607.7	660.0	+
Xiangtan	Southcentral	290	51.0	232.7	236.3	+
Yancheng	East	831	50.7	673.3	706.0	+
Haikou	Southcentral	167	49.0	79.3	75.3	–
Zibo	East	432	47.3	716.3	726.3	+
Taiyuan	North	370	43.7	121.3	131.3	+
Taian	East	569	43.3	351.3	375.3	+
Yangzhou	East	462	40.3	617.7	634.7	+
Tangshan	North	760	34.7	319.3	328.0	+
Quzhou	East	257	34.0	172.7	182.3	+
Guangyuan	Southwestern	305	33.0	66.0	62.7	–
Shantou	Southcentral	559	32.7	354.7	401.3	+
Baoding	North	1207	32.3	353.0	401.0	+
Zhuzhou	Southcentral	404	31.7	345.3	355.7	+
Yichang	Southcentral	394	31.3	340.7	304.7	–
Lanzhou	Northwestern	324	30.7	123.3	123.7	+
Nanyang	Southcentral	1195	30.3	414.0	487.7	+
Hengyang	Southcentral	799	30.0	180.7	178.7	–
Zhangzhou	East	508	27.0	529.0	506.7	–
Dongying	East	193	26.3	245.7	239.7	–
Linyi	East	1141	26.0	1014.3	1055.7	+
Chuzhou	East	454	25.3	279.0	264.7	–
Ganzhou	East	971	25.0	259.7	279.3	+
Deyang	Southwestern	392	24.3	273.3	319.3	+
Pingxiang	East	200	24.0	395.0	515.0	+
Xuchang	Southcentral	510	24.0	305.7	344.3	+
Xiaogan	Southcentral	523	24.0	231.0	230.7	–
Xinxiang	Southcentral	646	23.0	254.7	218.0	–
Heze	East	1015	22.0	755.7	808.3	+
Baotou	North	224	21.7	185.7	178.0	–
Handan	North	1055	21.0	237.7	247.7	+
Luzhou	Southwestern	508	20.7	118.7	123.7	+
Huaian	East	568	20.3	514.3	552.3	+
Longyan	East	314	20.0	216.3	242.7	+
Rizhao	East	300	20.0	155.3	174.0	+
Yueyang	Southcentral	571	19.7	245.7	251.7	+
Luan	East	587	19.0	175.0	168.0	–
Dezhou	East	593	18.3	712.3	741.0	+
Qinhuangdao	North	298	18.3	93.0	97.0	+
Langfang	North	470	18.3	241.3	220.7	–
Suqian	East	592	17.7	502.7	508.3	+
Nanping	East	321	17.3	196.0	214.3	+
Hohhot	North	241	16.3	110.0	133.7	+
Yichun1 (Jiangxi)	East	602	16.0	41.0	41.7	+
Yinchuan	Northwestern	184	15.3	164.3	134.3	–
Jiaozuo	Southcentral	374	15.0	302.0	285.3	–
Lishui	East	268	15.0	216.7	198.0	–
Zhanjiang	Southcentral	835	14.7	133.7	151.7	+
Xining	Northwestern	203	14.7	97.7	94.3	–
Xianyang	Northwestern	530	14.0	193.7	221.7	+
Zaozhuang	East	413	14.0	265.3	288.3	+
Zhaoqing	Southcentral	444	13.3	197.0	194.0	–
Binzhou	East	392	13.3	265.3	254.3	–
Huaibei	East	217	13.3	144.3	121.0	–
Zhoushan	East	97	13.0	251.7	268.0	+
Jilin	Northeastern	422	12.7	216.3	226.0	+
Guang'an	Southwestern	467	12.7	75.0	82.0	+
Xuancheng	East	280	12.7	252.3	255.0	+
Fushun	Northeastern	215	12.3	71.7	71.3	–
Liaocheng	East	633	12.3	550.0	535.7	–
Xingtai	North	788	12.0	250.7	267.7	+
Fuyang	East	1062	12.0	266.0	266.7	+
Kaifeng	Southcentral	559	11.7	245.7	286.0	+
Shaoyang	Southcentral	830	11.7	148.3	155.3	+
Laiwu	East	129	11.3	448.3	584.3	+
Ningde	East	352	11.3	209.7	218.0	+
Anshun	Southwestern	300	11.0	57.3	67.3	+
Jingzhou	Southcentral	646	11.0	226.3	231.3	+
Baishan	Northeastern	122	11.0	84.0	88.3	+
Daqing	Northeastern	276	11.0	116.3	119.3	+
Yulin1 (Guangxi)	Southcentral	717	10.7	77.3	79.0	+
Huainan	East	389	10.7	118.7	114.7	–
Baise	Southcentral	417	10.3	53.7	69.3	+
Qiqihar	Northeastern	544	10.0	64.0	66.3	+
Xinyang	Southcentral	908	9.3	198.7	207.3	+
Huaihua	Southcentral	523	9.3	108.3	96.0	–
Shuozhou	North	163	9.0	72.3	66.7	–
Chaoyang	Northeastern	341	9.0	50.0	43.3	–
Hengshui	North	455	8.7	171.0	212.7	+
Putian	East	350	8.3	222.0	272.3	+
Xinyu	East	124	8.3	96.3	117.0	+
Bengbu	East	380	8.3	222.3	226.7	+
Siping	Northeastern	324	8.3	130.7	114.7	–
Zhoukou	Southcentral	1259	8.3	198.7	165.0	–
Changde	Southcentral	611	8.0	178.7	204.7	+
Loudi	Southcentral	453	8.0	128.7	114.3	–
Bozhou	East	647	7.7	151.0	155.3	+
Meishan	Southwestern	350	7.7	103.3	98.3	–
Shaoguan	Southcentral	334	7.3	95.0	118.3	+
Sanmenxia	Southcentral	229	6.7	135.7	157.3	+
Anyang	Southcentral	626	6.7	190.3	206.7	+
Yiyang	Southcentral	484	6.7	151.0	163.0	+
Yulin2 (Shaanxi)	Northwestern	382	6.7	170.7	179.7	+
Heyuan	Southcentral	373	6.7	85.0	87.7	+
Qinzhou	Southcentral	409	6.7	58.3	58.7	+
Panzhihua	Southwestern	111	6.7	87.3	85.7	–
Jinzhou	Northeastern	302	6.7	103.0	97.0	–
Yibin	Southwestern	556	6.7	118.7	112.3	–
Chifeng	North	463	6.7	123.3	99.0	–
Chaozhou	Southcentral	274	6.3	142.0	160.0	+
Zhumadian	Southcentral	949	6.3	255.3	267.0	+
Puyang	Southcentral	433	6.0	176.7	191.0	+
Jingdezhen	East	169	6.0	88.7	102.7	+
Jinzhong	North	332	6.0	132.0	137.3	+
Luohe	Southcentral	269	6.0	129.0	133.3	+
Shuizuishan	Northwestern	75	6.0	76.7	73.0	–
Nanchong	Southwestern	741	6.0	122.7	107.0	–
Tiding	Northeastern	300	5.7	33.3	40.3	+
Guigang	Southcentral	555	5.7	54.3	57.3	+
Yaan	Southwestern	155	5.7	95.7	75.7	–
Tonghua	Northeastern	220	5.3	110.3	128.0	+
Yongzhou	Southcentral	645	5.3	124.0	130.3	+
Baoji	Northwestern	384	5.3	119.3	123.7	+
Huangshi	Southcentral	270	5.0	147.7	171.0	+
Chengde	North	383	5.0	85.3	92.0	+
Wuzhong	Northwestern	142	5.0	75.0	70.0	–
Dandong	Northeastern	238	5.0	72.7	59.7	–
Jiujiang	East	520	5.0	281.0	264.0	–
Shangqiu	Southcentral	977	5.0	238.3	210.7	–
Benxi	Northeastern	150	4.7	367.0	498.3	+
Beihai	Southcentral	174	4.7	225.7	308.7	+
Jieyang	Southcentral	697	4.7	343.3	387.3	+
Shiyan	Southcentral	348	4.7	191.0	203.7	+
Yuxi	Southwestern	217	4.7	92.3	103.3	+
Lasa	Southwestern	54	4.7	58.7	69.3	+
Tongliao	North	319	4.7	122.0	128.3	+
Ziyang	Southwestern	355	4.7	68.3	67.7	–
Panjin	Northeastern	130	4.7	97.7	88.7	–
Suining	Southwestern	378	4.7	124.3	96.7	–
Zunyi	Southwestern	802	4.7	203.3	171.0	–
Jingmen	Southcentral	300	4.3	223.3	247.0	+
Zhangjiakou	North	470	4.3	99.7	112.7	+
Wuzhou	Southcentral	347	4.3	65.3	72.0	+
Fuzhou2 (Jiangxi)	East	401	4.3	170.7	175.0	+
Cangzhou	North	780	4.0	484.3	516.0	+
Mudanjiang	Northeastern	259	4.0	96.7	118.3	+
Meizhou	Southcentral	551	4.0	58.3	71.0	+
Pingdingshan	Southcentral	568	4.0	169.0	181.3	+
Datong	North	318	4.0	56.3	55.3	–
Haidong	Northwestern	171	4.0	39.0	28.0	–
Jian	East	535	4.0	205.3	190.3	–
Yingkou	Northeastern	233	3.7	105.7	132.0	+
Xiangyang	Southcentral	594	3.7	368.0	388.0	+
Fuxin	Northeastern	189	3.7	32.7	42.0	+
Tongling	East	171	3.7	127.7	131.0	+
Qingyuan	Southcentral	432	3.7	111.0	105.0	–
Zhongwei	Northwestern	121	3.7	51.3	38.0	–
Zhangye	Northwestern	131	3.3	65.3	72.7	+
Chenzhou	Southcentral	535	3.3	186.7	188.7	+
Chongzuo	Southcentral	251	3.3	55.0	53.0	–
Yangjiang	Southcentral	296	3.3	122.3	114.7	–
Sanming	East	287	3.3	382.0	370.0	–
Huangshan	East	148	3.0	121.0	115.7	–
Xianning	Southcentral	304	3.0	145.7	136.7	–
Huanggang	Southcentral	747	3.0	228.0	208.0	–
Shangrao	East	782	2.7	182.3	195.7	+
Jiuquan	Northwestern	112	2.7	67.3	77.7	+
Liaoyang	Northeastern	179	2.7	62.3	56.7	–
Hechi	Southcentral	429	2.7	38.7	32.7	–
Hulunbuir	North	259	2.3	103.0	107.0	+
Neijiang	Southwestern	420	2.3	81.3	80.3	–
Anshan	Northeastern	346	2.3	154.3	150.0	–
Zhangjiajie	Southcentral	470	2.3	57.3	53.0	–
Jiamusi	Northeastern	238	2.3	62.0	55.7	–
Bazhong	Southwestern	375	2.3	41.7	35.0	–
Yuncheng	North	531	2.3	79.7	66.3	–
Suzhou2 (Anhui)	East	654	2.0	1933.0	2602.7	+
Laibin	Southcentral	269	2.0	37.7	56.3	+
Jixi	Northeastern	181	2.0	38.0	47.3	+
Jinchang	Northwestern	46	2.0	41.7	44.0	+
Baiyin	Northwestern	182	2.0	43.3	40.7	–
Bayannur	North	175	2.0	85.3	80.7	–
Jincheng	North	220	2.0	75.3	68.3	–
Suizhou	Southcentral	252	2.0	99.7	89.7	–
Bijie	Southwestern	917	2.0	82.7	61.3	–
Yunfu	Southcentral	301	1.7	139.7	149.0	+
Dazhou	Southwestern	684	1.7	67.0	70.0	+
Leshan	Southwestern	355	1.7	124.7	126.7	+
Qujing	Southwestern	653	1.7	105.7	107.7	+
Yangquan	North	133	1.7	28.7	30.7	+
Tianshui	Northwestern	371	1.7	29.7	27.0	–
Suihua	Northeastern	543	1.7	58.0	52.0	–
Changzhi	North	339	1.3	68.3	89.3	+
Ulanqab	North	274	1.3	61.7	72.3	+
Tongren	Southwestern	441	1.3	79.0	84.7	+
Xinzhou	North	308	1.3	69.0	71.7	+
Songyuan	Northeastern	278	1.3	134.3	136.7	+
Shanwei	Southcentral	362	1.3	48.0	46.0	–
Liupanshui	Southwestern	340	1.3	79.3	77.0	–
Hezhou	Southcentral	243	1.3	39.3	35.0	–
Longnan	Northwestern	288	1.3	22.0	16.0	–
Yingtan	East	128	1.3	81.3	73.7	–
Baicheng	Northeastern	193	1.3	81.0	71.7	–
Hanzhong	Northwestern	384	1.3	90.7	79.7	–
Ankang	Northwestern	304	1.3	105.0	87.3	–
Jiangmen	Southcentral	394	1.0	420.7	432.0	+
Puer	Southwestern	251	1.0	47.7	58.3	+
Weinan	Northwestern	557	1.0	87.0	93.0	+
Lijiang	Southwestern	122	1.0	28.7	34.0	+
Chizhou	East	162	1.0	106.7	111.3	+
Linfen	North	434	1.0	40.0	40.0	–
Qitaihe	Northeastern	80	1.0	31.3	30.3	–
Ezhou	Southcentral	111	0.7	372.7	558.0	+
Baoshan	Southwestern	261	0.7	122.7	140.0	+
Lincang	Southwestern	237	0.7	64.0	80.3	+
Fangchenggang	Southcentral	97	0.7	58.7	66.3	+
Dingxi	Northwestern	303	0.7	24.3	26.7	+
Hebi	Southcentral	170	0.7	114.7	117.0	+
Heihe	Northeastern	163	0.7	34.0	35.0	+
Shuangyashan	Northeastern	145	0.7	46.7	41.7	–
Shangluo	Northwestern	253	0.3	50.0	57.3	+
Liaoyuan	Northeastern	120	0.3	73.7	76.7	+
Hegang	Northeastern	104	0.3	37.0	39.3	+
Wuhai	North	44	0.3	72.3	73.0	+
Yanan	Northwestern	237	0.3	59.7	57.7	–
Pingliang	Northwestern	234	0.3	25.7	23.3	–
Sanya	Southcentral	58	0.3	43.0	39.7	–
Huludao	Northeastern	280	0.3	53.3	45.0	–
Wuwei	Northwestern	191	0.3	55.7	46.0	–
Yichun2 (Heilongjiang)	Northeastern	602	0.3	164.0	129.0	–

The number 1 and 2 indicates different cities as they have the same English spelling

**Table 3:** Calibrated external system parameters

Parameter	Description	Type	Calibrated value
*BasePatentProb*	Scaling parameter for patent probability (originally estimated econometrically)	$\left[ {0,1} \right] \in {\mathbb{R}}_{0}$	0.1
*CollabInternalProb*	Share of agents performing collaborative research (vs. internal research)	$\left[ {0,1} \right] \in {\mathbb{R}}_{0}$	0.4
*CollabMemorySize*	Length of collaboration memory vector (determines number of former collaboration partners being remembered)	$\left[ {1,10} \right] \in {\mathbb{N}}$	5
*CollabModeProb*	Share of agents with service-oriented mode of collaboration (vs. research-mode)	$\left[ {0,1} \right] \in {\mathbb{R}}_{0}$	0.4
*ResearchStrategyProb*	Share of agents with exploitative mode of knowledge creation (vs. explorative)	$\left[ {0,1} \right] \in {\mathbb{R}}_{0}$	0.3
*Lambda* $\left[ \lambda \right]$	Determines the degree of radicality in search for a research target technology class (in exploitative research)	$\left[ {0,1} \right] \in {\mathbb{R}}_{0}$	0.7

Additionally, a second set of external system parameters (Table 4) is purely specified by means of user input (i.e. they are not calibrated and not empirically based)

**Table 4:** Non-calibrated external system parameters

Parameter	Description	Type	Initialisation value
*ExplorativePathLength*	Indicates the maximum length of research project for explorative mode of knowledge creation (1 step = 1 month)	${\text{unif}}\left( {0,x} \right),{ }x \in {\mathbb{R}}_{ + }$	5
*Delta* $\left[ \delta \right]$	Determines how much is learnt from the partner in collaborative research (optimal learning distance)	${\mathbb{R}}_{ + }$	1

## Appendix B: City-specific patent profiles

See Figs. 9, 10, 11, 12.

## Appendix C: IPC classes

See Table 5.

**Table 5:** List of relevant IPC classes—in alphabetical order (from Fig. 7, IPC classes with more than 1000 simulated patents are displayed)

Code	Description
A23N	Foods or foodstuffs
B06B	Generating or transmitting mechanical vibrations
B07B	Separating solids from solids by sieving, screening, sifting or by using gas currents
B23P	Metal-working not otherwise provided for
B23Q	Details, components, or accessories for machine tools
B26B	Hand-held cutting tools
B26F	Perforating; punching; cutting-out; stamping-out; severing by means other than cutting
B28B	Shaping clay or other ceramic com + itions
B60J	Windows, windscreens, non-fixed roofs, doors, or similar devices for vehicles
B60L	Propulsion of electrically-propelled vehicles
B60Q	Arrangement of signalling or lighting devices for vehicles
B60R	Vehicles, vehicle fittings, or vehicle parts
B60T	Vehicle brake control systems; brake control systems
B60W	Conjoint control of vehicle sub-units of different type or different function
B65G	Transport or storage devices
C03B	Manufacture or shaping of glass, or of mineral or slag wool
C03C	Chemical com + ition of glasses, glazes or vitreous enamels; surface treatment of glass
C04B	Lime; magnesia; slag; cements
C14B	Mechanical treatment or processing of skins, hides, or leather in general
D06H	Marking, inspecting, seaming or severing textile materials
E05B	Locks; accessories therefor; handcuffs
E05C	Bolts or fastening devices for wings, specially for doors or windows
E05D	Hinges or suspension devices for doors, windows or wings
E05F	Devices for moving wings into open or closed + ition
E06B	Fixed or movable closures for openings in buildings, vehicles, fences, or like enclosures
F01D	Non- + itive-displacement machines or engines
F01K	Steam engine plants; steam accumulators; engine plants
F02C	Gas-turbine plants; air intakes for jet-propulsion plants
F04D	Non- + itive-displacement pumps
F16F	Springs; shock-absorbers; means for damping vibration
F16M	Frames, casings or beds of engines, machines or apparatus
F25B	Refrigeration machines; combined heating and refrigeration systems; heat pump systems
F25C	Producing, working or handling ice
F25D	Refrigerators; cold rooms; ice-boxes; cooling or freezing apparatus
G01F	Measuring volume, volume flow, mass flow, or liquid level
G01L	Measuring force, stress, torque, work, mechanical power, mechanical efficiency, or fluid pressure
G03B	Apparatus or arrangements for taking photographs or for projecting or viewing them
G06F	Electric digital data processing
G06K	Graphical data reading; presentation of data; record carriers
G06T	Image data processing or generation
G07B	Ticket-issuing apparatus; taximeters; arrangements or apparatus for collecting fares
G07C	Time or attendance registers; voting or lottery apparatus
G08B	Signalling or calling systems; order telegraphs; alarm systems
G08G	Traffic control systems
G10C	Pianos, harpsichords, spinets or similar stringed musical instruments with one or more keyboards
G10D	Stringed musical instruments; wind musical instruments; accordions or concertinas; percussion musical instruments
G10G	Representation of music; recording music in notation form; accessories for music or musical instruments
G10H	Electrophonic musical instruments; instruments in which the tones are generated by electromechanical means or electronic generators
H01M	Processes or means, e.g. batteries, for the direct conversion of chemical energy into electrical energy
H02H	Emergency protective circuit arrangements
H02J	Circuit arrangements or systems for supplying or distributing electric power; systems for storing electric energy
H02K	Dynamo-electric machines
H02M	Conversion of electric power
H02P	Control or regulation of electric motors, electric generators or dynamo-electric converters
H04L	Transmission of digital information
H04N	Pictorial communication
H04W	Wireless communication networks

## Footnotes

1 State Council of the PRC, Opinions on Establishing a New Mechanism for More Effective Regional Coordinated Development, http://www.gov.cn/zhengce/2018-11/29/content_5344537.htm, November 2018.

2 While many ABMs certainly pay attention to a thorough theoretical foundation and transparent presentation and documentation (e.g. see Vermeulen and Paier 2017 for examples), only comparably few studies have a particularly empirical focus, strengthening the model’s validity and its usability in a policy context.

3 The characteristics sector, size, and R&D intensity are deducted from region and industry characteristics, since no organisational-level data is available. Importantly, agents need to be representative for each region with respect to its characteristics (see Sect. 5.1 for details on agent initialisation).

4 Incopat is a patent database provider from China with a collection of more than 100 million patent documents worldwide. https://www.incopat.com.

5 The technology space is defined as a network comprising a set of technology classes, with the links indicating the technological proximity between these classes.

6 The sample of Chinese regions is determined by the availability of empirical data drawn from the China city statistical yearbook.

7 The degree centrality is chosen as a simple but yet informative and clearly graspable ‘quality’ aspect to reflect the regions’ embeddedness in the knowledge network in terms of their number of collaborations with other regions.

8 While knowledge gain is the direct model’s output, patents are derived after the simulation as an additional well-known measure of knowledge output that allows also for better comparability with other studies investigating regional knowledge creation. Due to the lack of organisation-level data, the regression model is estimated on the city level. We estimate a Poisson regression model to account for the true integer nature and the distributional assumptions of the number of patents as the dependent variable.

9 Additionally, considerations on the mechanisms of scientific project work and patenting that determine the periodicities and scheduling in the knowledge creation process were taken into account when developing the model.

10 The map covers the study area only, not the whole territory of China.

11 One run takes about 12 h, i.e. we face limitations to experiment with more time steps. We have chosen 120 steps since this is sufficient to illustrative the trend of changes.

12 Ranks proved to be better suited due to the strong skewness of the data (with a few outliers); using ranks reduces the influence of outliers and overall reduces the sensitivity to the skewed distribution increasing the robustness of the results (also when comparing different time periods). Moreover, ranks allow to better emphasise relative improvements or declines which is beneficial to illustrating regional convergence processes.

13 Chinese translation added in the list of regions in the Appendix.

14 To create the portfolio, the number of patents and the average growth rates (as for Fig. 4) are determined for both scenarios. Then, the mean of these two values over all cities is subtracted from each city value (‘demeaning’ the two variables for each city observation). Next, differences between these demeaned values of patents and growth rates between the baseline scenario and the scenario of increased collaborative research are determined: i) The ‘strength’ of the change is calculated by means of the Euclidean distance in the coordinate plot of the portfolio diagram; ii) The ‘direction’ of the change (positive or negative) is dependent on whether each city gained (positive) in their number of patents, their patent growth, or in both, when collaboration is increased. Demeaned logged number of patents = log⁡(patents)−mean(log⁡(patents)); demeaned average growth rate=average growth rate-mean(average growth rate); average growth rate = ((〖(patents〗_(t+1)- 〖patents〗_t))⁄(〖patents〗_t)*(1⁄(number of years))).

15 Shantou is located in the East of Guangzhou province and is one of the first special economic zones in China. It has a relatively open environment and also enjoyed the policy benefits of the reform and opening up earlier. Moreover, Shantou is fully grasping the advantages of cross-border communication and making effort to create a new highland for the digital economy.

16 Specialisation is measured by means of the Krugman Index of Specialisation (see Marelli 2007).
